# Glycolysis-related lncRNA TMEM105 upregulates LDHA to facilitate breast cancer liver metastasis via sponging miR-1208

**DOI:** 10.1038/s41419-023-05628-z

**Published:** 2023-02-03

**Authors:** Jinzhu Han, Xuyi Chen, Jianlong Wang, Bin Liu

**Affiliations:** 1 Department of Cancer, The Second Hospital of Hebei Medical University, Shijiazhuang, China; 2 Department of Neurosurgery, Characteristic Medical Center of Chinese People’s Armed Police Force, Tianjin, China; 3 Central Laboratory, The Second Hospital of Hebei Medical University, Shijiazhuang, China

**Keywords:** Breast cancer, Prognostic markers

## Abstract

Increased glycolysis is one of the key metabolic hallmarks of cancer cells. However, the roles of lncRNAs in energy metabolism and cancer metastasis remain unclear. Here, the expression of TMEM105 associated with glycolysis was dramatically elevated from normal to breast cancer to breast cancer liver metastasis tissues, and the survival analysis revealed that high TMEM105 expression was related to poor survival, especially in patients with liver metastasis. Moreover, TMEM105 facilitated the glycolysis of breast cancer cells and induced cell invasion and breast cancer liver metastasis (BCLM). Mechanistically, TMEM105 regulated LDHA expression by sponging miR-1208, which further promoted cell glycolysis and BCLM. Importantly, glycolytic production of lactate enhanced TMEM105 expression in breast cancer cells by activating the SHH-MAZ signaling pathway. These findings suggested that the lactate-responsive TMEM105 acted as a miRNA sponge, inducing BCLM via a glycolysis-mediated positive feedback loop, which might be a rational target for the treatment of BCLM patients.

## Introduction

Breast cancer is the most prevalent cancer in women. Although the improvement of early screening has led to great progress in the therapy of breast cancer, metastasis of cancer cells to other organs is the main influencing factor in the survival of the patients [1, 2]. The liver is a common site of breast cancer metastasis. Breast cancer liver metastasis (BCLM) has a poor prognosis, high mortality rate, and lack of effective therapy [3, 4]. Thus, it is necessary to clarify the key drivers and mechanisms of BCLM to improve the prognosis of the patients [5, 6].

Aerobic glycolysis also named the Warburg effect is an important characteristic of glucose metabolism in cancer cells [7]. The pattern of glucose metabolism in cancers differs from normal tissues. Cancer cells obtain the energy for survival through glycolysis even in the presence of sufficient oxygen, while activating multiple metabolism-related signaling pathways to promote cancer cell survival in harsh conditions [8, 9]. The elevated aerobic glycolysis disrupts NAD+ metabolism and alters the NADH/NAD+ redox ratio, resulting in the disruption of cellular redox homeostasis and facilitating cancer [10]. The significant role of glycolysis in cancers has been studied, but the role of glycolysis in BCLM and the underlying mechanisms are still not fully characterized.

The ncRNAs are not encoding proteins, but they influence gene expression at epigenetic, transcriptional, and post-transcriptional levels and are closely related to the incidence and prognosis of many diseases [11]. The increasing number of studies suggests that lncRNAs are extensively involved in cell proliferation, differentiation, apoptosis, invasion, and metastasis [12–14]. Moreover, it has been shown that lncRNAs are closely associated with cancer glycolysis [15, 16]. We previously reported that the lncRNA PCAT-1 enhances the oxygen-independent stability of HIF-1α, suggesting that lncRNAs play an important role in glucose metabolism in breast cancer cells [17]. Therefore, it is essential to clarify the specific molecular mechanisms involved in the regulation of glycolysis by lncRNAs, which could control phenotypic changes and serve as a novel therapeutic strategy for cancers [18].

In the present study, we identified that TMEM105 expression was dramatically increased from normal to breast cancer to breast cancer liver metastasis tissues, and high TMEM105 expression was related to poor survival, especially in patients with liver metastases. Mechanistically, TMEM105 regulated LDHA expression by sponging miR-1208, which further promoted glycolysis and BCLM. Moreover, glycolysis-generated lactate further upregulated TMEM105 through Sonic Hedgehog (SHH)-Myc-associated zinc finger protein (MAZ) signaling, which formed a positive feedback loop. Therefore, this signaling axis may be a potent and effective target for patients with BCLM.

## Results

### TMEM105 correlates with breast cancer liver metastasis

**Fig. 1:**
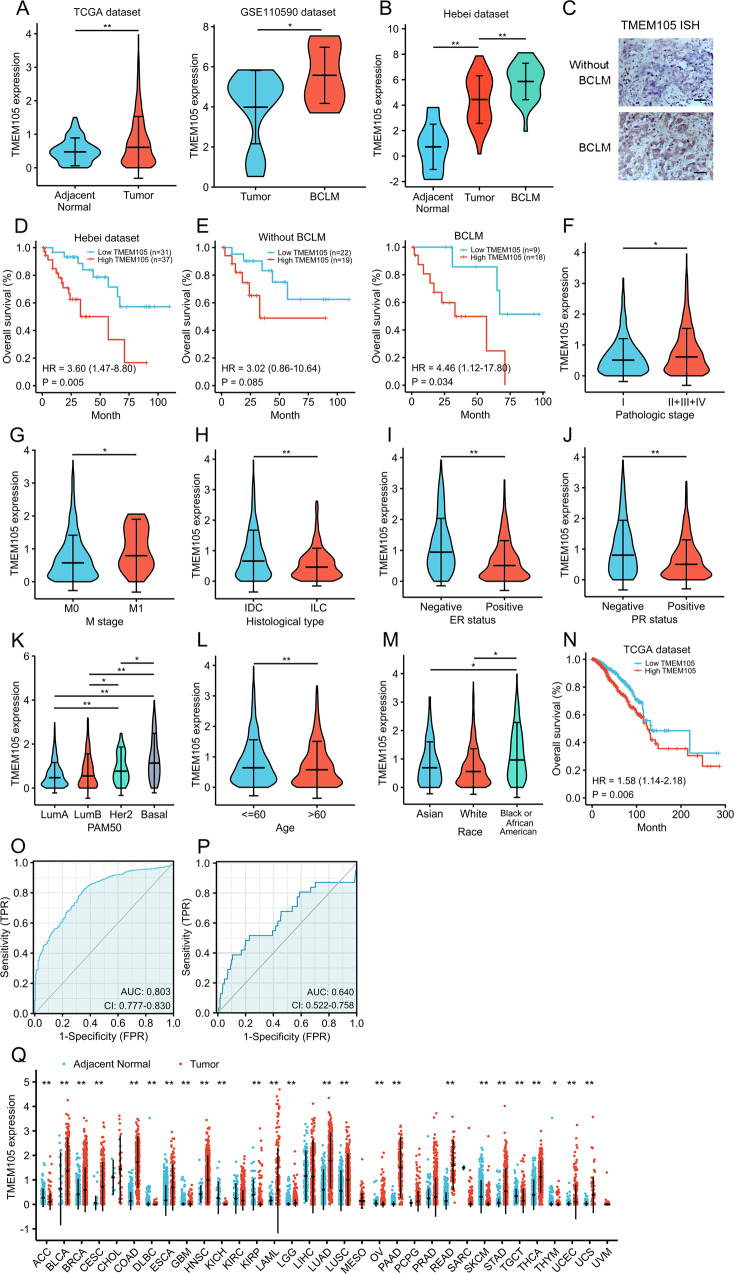
TMEM105 overexpression is correlated with poor prognosis and liver metastasis in breast cancer. **A** TMEM105 expression in adjacent normal, breast cancer, and BCLM tissues in the TCGA and GSE110590 datasets. **B** TMEM105 expression in adjacent normal tissues, breast cancer, and paired BCLM in the Hebei dataset (*n* = 68). **C** Representative ISH detection for TMEM105 expression in breast cancer patients without or with liver metastasis. Scale bars, 50 μm. **D** Kaplan–Meier analysis for OS according to TMEM105 expression in the Hebei dataset. **E** Kaplan–Meier analysis for OS according to TMEM105 expression of the patients without or with BCLM in the Hebei dataset. The median TMEM105 expression was used as the cutoff value. TCGA dataset showed that TMEM105 overexpression correlated with pathological grade (**F**), distant metastasis (**G**), histological type (**H**), ER status (**I**), PR status (**J**), PAM50 subtype (**K**), age (**L**), and race (**M**) in the breast cancer patients. **N** Kaplan–Meier analysis for OS of breast cancer patients according to TMEM105 expression in the TCGA dataset. **O** The ROC curve for predicting breast cancer depended on TMEM105 expression based on the TCGA dataset. **P** The ROC curve for predicting breast cancer metastasis depended on TMEM105 expression based on the TCGA dataset. **Q** Analysis of the TCGA dataset revealed that TMEM105 was highly expressed in different cancer tissues relative to the normal tissues. *p < 0.05, **p < 0.01.

To identify the changes of lncRNAs in BCLM samples relative to primary breast cancer and normal tissues, we analyzed the high-throughput sequencing data from two independent cohorts. The first cohort was from GSE110590 collecting 12 primary breast cancer and 14 BCLM patient samples [19]. The second cohort was from the Cancer Genome Atlas (TCGA) dataset including 1091 breast cancer samples and 113 adjacent normal breast samples. It indicated that TMEM105 expression was higher in cancer tissues with BCLM than in the cancer tissues without BCLM (Fig. 1A). To further validate the findings from the analysis of the public datasets, we detected the expression of TMEM105 by qRT-PCR in the specimens from breast cancer patients collected in our institution. Our results revealed that TMEM105 expression was increased from adjacent normal tissues to breast cancer to the paired BCLM (Fig. 1B). ISH analysis showed that TMEM105 expression was slightly increased in breast cancer tissues without BCLM, but intensely upregulated in BCLM tissues (Fig. 1C). The overall survival (OS) time was shorter in TMEM105 high expressing breast cancer patients by survival analysis (Fig. 1D). In particular, although patients with high TMEM105 expression exhibited a tendency to have shorter OS times in both BCLM and without BCLM, the high TMEM105 expression showed significantly lower OS only in the BCLM patients (Fig. 1E). Thus, TMEM105 played an important role in the progression of breast cancer, especially during BCLM. Analysis of the TCGA dataset showed that TMEM105 expression was strongly correlated with pathological grade, distant metastasis, histological type, estrogen receptor (ER) status, progesterone receptor (PR) status, PAM50 subtype, age, and race (Fig. 1F–M). Importantly, the TCGA dataset indicated that breast cancer patients with high TMEM105 expression had poor OS (Fig. 1N). The ROC curve analysis based on the TCGA dataset was performed to evaluate the diagnostic effect of TMEM105 expression. The area under the ROC curve (AUC) was 0.803 (95% CI = 0.777–0.830) (Fig. 1O), indicating a high diagnostic value of TMEM105 expression in breast cancer. To clarify whether TMEM105 could be used as a predictor of metastasis, we defined patients in the TCGA dataset according to the occurrence of distant metastasis, and the results showed that TMEM105 significantly predicted breast cancer metastasis using ROC curve analysis (AUC = 0.640, Fig. 1P). Moreover, similar to the prognostic values of pathologic stage (II + III + IV versus I), tumor size (T3 + T4 versus T1 + T2), lymph node metastasis (N2-3 versus N0-1), M stage (M1 versus M0), age (>60 versus ≤60), PAM50 (LumB versus LumA), and PAM50 (Her2 versus LumA), the univariate regression analysis revealed that high TMEM105 expression was another effective predictive factor for poor OS (HR = 1.575, *p* = 0.006, Table 1). To further validate the prognostic value of TMEM105 expression, multivariate analysis was used to identify risk assessments associated with OS. Notably, TMEM105 expression was an independent predictor for OS (HR = 1.483, *p* = 0.047, Table 1).

**Table 1:** Univariate and multivariate Cox regression analyses of overall survival in breast cancer patients.

Characteristics	Univariate analysis		Multivariate analysis	
	Hazard ratio (95% CI)	*p* value	Hazard ratio (95% CI)	*p* value
Pathologic stage (II + III + IV versus I)	2.210 (1.313–3.721)	0.003	1.925 (1.047–3.542)	0.035
Tumor size (T3 + T4 versus T1 + T2)	1.608 (1.110–2.329)	0.012	1.332 (0.827–2.147)	0.238
Lymph node metastasis (N2-3 versus N0-1)	2.163 (1.472–3.180)	<0.001	1.992 (1.211–3.277)	0.007
M stage (M1 versus M0)	4.254 (2.468–7.334)	<0.001	2.114 (0.970–4.607)	0.060
Race (White versus Asian)	1.325 (0.420–4.186)	0.631		
Race (Black + African American versus Asian)	1.525 (0.463–5.024)	0.488		
Histological type (ILC versus IDC)	0.827 (0.526–1.299)	0.410		
ER status (Positive versus Negative)	0.725 (0.505–1.041)	0.082	0.623 (0.310–1.250)	0.183
PR status (Positive versus Negative)	0.733 (0.525–1.025)	0.070	0.780 (0.413–1.474)	0.445
Age (>60 versus ≤60)	2.020 (1.465–2.784)	<0.001	2.174 (1.475–3.205)	<0.001
PAM50 (LumB versus LumA)	1.663 (1.088–2.541)	0.019	1.080 (0.653–1.787)	0.765
PAM50 (Her2 versus LumA)	2.261 (1.325–3.859)	0.003	1.287 (0.585–2.831)	0.530
PAM50 (Basal versus LumA)	1.285 (0.833–1.981)	0.257	0.845 (0.394–1.809)	0.664
TMEM105 expression (high versus low)	1.575 (1.138–2.180)	0.006	1.483 (1.005–2.186)	0.047

To assess the clinical significance of TMEM105 in the Hebei dataset, we next investigated the relationship between TMEM105 expression and clinicopathological characteristics. High TMEM105 expression was notably related to the pathological grade, distant metastasis, histological type, ER status, PR status, and PAM50 subtype (Supplementary Fig. 1A–G). Moreover, time-dependent ROC curve analysis was constructed to assess the diagnostic accuracy, sensitivity, and specificity of TMEM105 at OS. It showed the 2-year AUC was 0.711, the 3-year AUC was 0.753, and the 5-year AUC was 0.778 (Supplementary Fig. 1H). Univariate and multivariate Cox proportional hazards analyses revealed that TMEM105 expression was an independent prognostic factor for OS in breast cancer patients in the Hebei dataset (Supplementary Table S1).

Furthermore, analysis of the TCGA dataset showed that TMEM105 was notably overexpressed in several types of human cancers, such as bladder urothelial carcinoma, esophageal carcinoma, and many other types of cancers (Fig. 1Q). Moreover, high TMEM105 expression was associated with cancer metastasis and poor prognosis (Supplementary Fig. 2A–P), which suggested that TMEM105 might have a carcinogenic role in the progression and development of human cancers.

### TMEM105 augments glycolysis by promoting LDHA expression in breast cancer cells

**Fig. 2:**
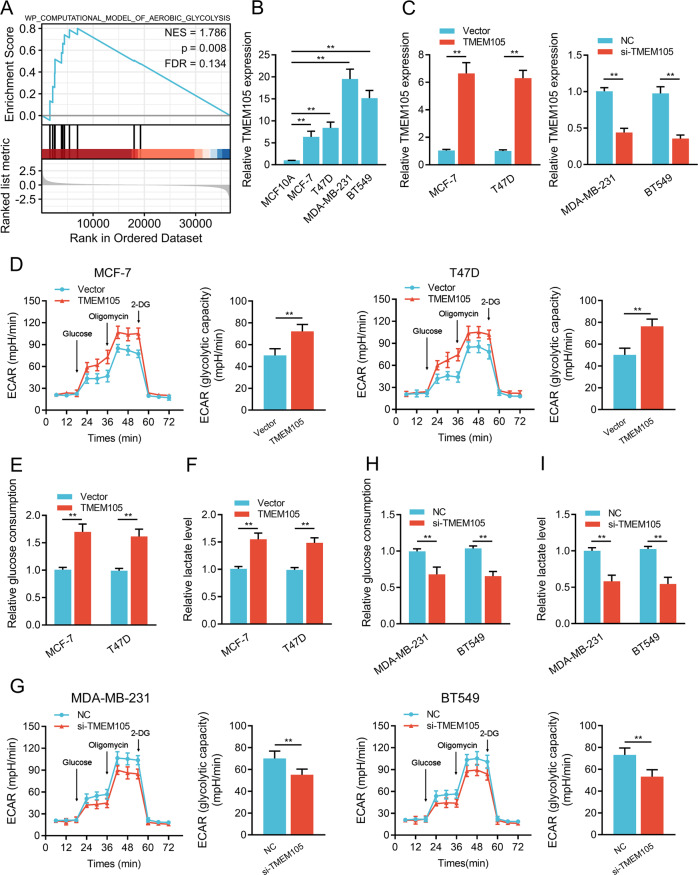
TMEM105 facilitates glycolysis in breast cancer cells. **A** GSEA analysis showed the different gene set between TMEM105-high and TMEM105-low of breast cancer patients in the TCGA dataset. NES normalized enrichment score, FDR false discovery rate. **B** Expression of TMEM105 was determined by qRT-PCR in MCF10A, MCF-7, T47D, MDA-MB-231, and BT549 cells. **C** Effects of overexpression or knockdown of TMEM105 in breast cancer cells as shown by qRT-PCR. ECAR level (**D**), glucose consumption (**E**), and lactate production (**F**) were determined after TMEM105 overexpression in MCF-7 and T47D cells. ECAR level (**G**), glucose consumption (**H**), and lactate production (**I**) were measured after the knockdown of TMEM105 in MDA-MB-231 and BT549 cells. 2-DG 2-deoxyglucose. **p < 0.01.

To clarify the potential function of TMEM105, we performed gene set enrichment analysis (GSEA) on the data of breast cancer patients in the TCGA dataset. The significantly enriched gene set in the samples with high TMEM105 expression was aerobic glycolysis (FDR = 0.134, *p* = 0.008) (Fig. 2A), suggesting an important role of TMEM105 in the regulation of gene expression of aerobic glycolysis. Furthermore, we evaluated the expression of TMEM105 in breast cancer cells and normal breast epithelial cell MCF10A. The results showed that the TMEM105 expression in breast cancer cells was significantly higher compared to MCF10A cells (Fig. 2B). Among the breast cancer cells, TMEM105 expression was lower in MCF-7/T47D and higher in MDA-MB-231/BT549 cells, so these cell lines were used to prepare TMEM105 overexpression or knockdown cells for further studies (Fig. 2C). Moreover, overexpression or knockdown of TMEM105 promoted or inhibited extracellular acidification rate (ECAR), glucose consumption, and lactate production in MCF-7/T47D or MDA-MB-231/BT549 cells, respectively (Fig. 2D–I). These data suggested that TMEM105 was involved in regulating glycolysis in breast cancer cells.

**Fig. 3:**
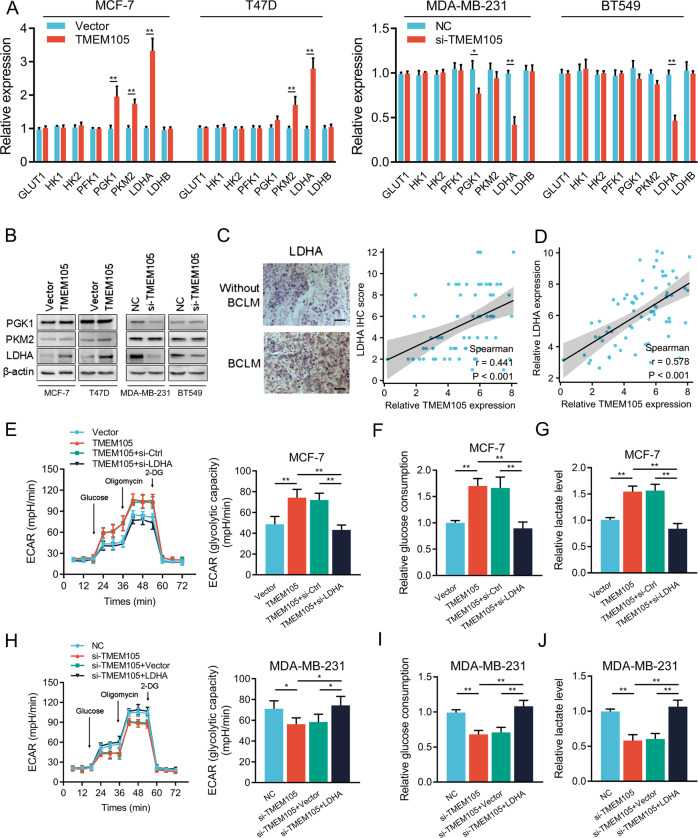
TMEM105 enhances glycolysis in breast cancer cells by promoting LDHA expression. **A** Expression of glycolysis-related genes in TMEM105-overexpressing MCF-7 and T47D cells as well as in TMEM105-knockdown MDA-MB-231 and BT549 cells. **B** Western blot analysis of PGK1, PKM2, and LDHA in TMEM105-overexpressing MCF-7 and T47D cells as well as in TMEM105-knockdown MDA-MB-231 and BT549 cells. **C** Representative immunohistochemical staining for LDHA expression in breast cancer tissues without or with BCLM. Spearman’s correlation between TMEM105 and LDHA expression in breast cancer tissues of the patients in the Hebei dataset (*n* = 68). Scale bars, 50 μm. **D** The correlation between TMEM105 and LDHA expression determined by qRT-PCR in breast cancer tissues in the Hebei dataset. ECAR level (**E**), glucose consumption (**F**), and lactate production (**G**) were determined in TMEM105-overexpressing MCF-7 cells. ECAR level (**H**), glucose consumption (**I**), and lactate production (**J**) were determined in TMEM105-knockdown MDA-MB-231 cells. *p < 0.05, **p < 0.01.

In cancer development, the key regulators of the glycolytic enzyme consist of glucose transporter 1 (GLUT1), hexokinase1/2 (HK1/2), phosphofructokinase 2/fructose bisphosphatase 3 (PFK2/PFKFP3), phosphoglycerate kinase 1 (PGK1), pyruvate kinase 2 (PKM2), and lactate dehydrogenase (LDH). Our results showed that overexpression or knockdown of TMEM105 promoted or inhibited the mRNA expression of PGK1, PKM2, and LDHA, respectively (Fig. 3A). Western blot analysis further showed that the LDHA expression was remarkably enhanced or reduced after overexpression or knockdown of TMEM105, while no notable changes were detected for PGK1 and PKM2 expression (Fig. 3B). Additionally, quantitative analysis of LDHA expression in breast cancer samples showed that LDHA expression was positively related to TMEM105 expression, particularly in BCLM tissues (Fig. 3C, D). Furthermore, LDHA knockdown or overexpression in TMEM105-overexpressing or knockdown cells partially blocked the promotion or inhibition of ECAR, glucose consumption, and lactate production (Fig. 3E–J). The above findings indicated that TMEM105 enhanced the glycolysis of breast cancer cells through LDHA.

### TMEM105 mediates breast cancer cell invasion and BCLM via upregulating LDHA

**Fig. 4:**
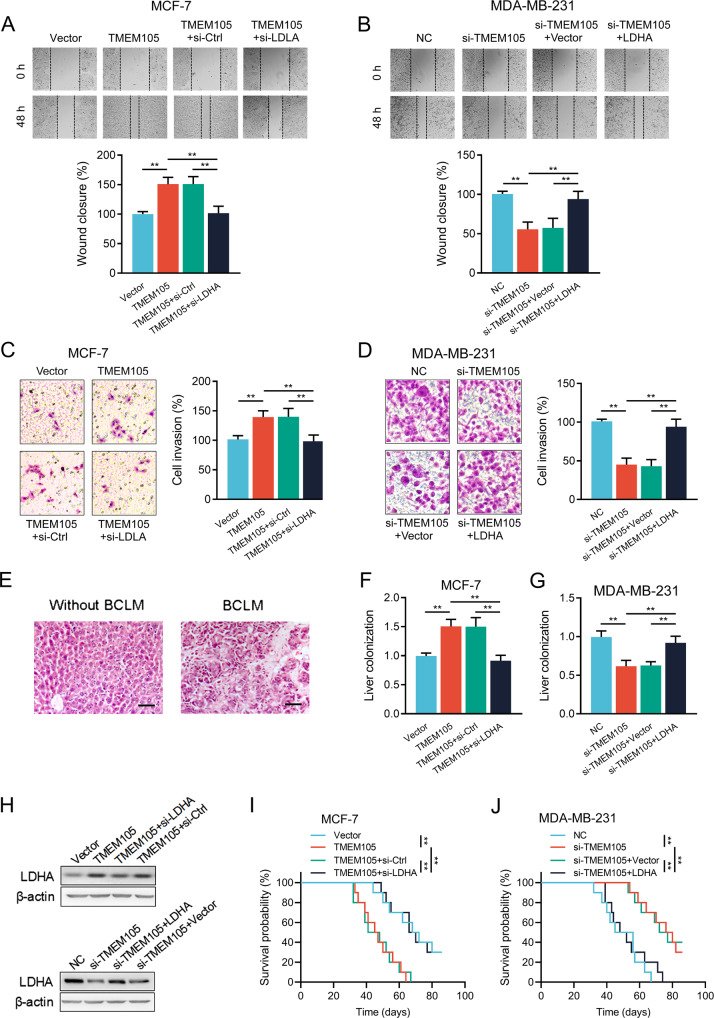
TMEM105 enhances breast cancer cell invasion and liver metastasis via promoting LDHA expression. Wound healing assay was performed to detect the effects of LDHA knockdown or overexpression on the migration of TMEM105-overexpressing MCF-7 cells (**A**) and TMEM105-knockdown MDA-MB-231 cells (**B**). Transwell matrigel assay was performed to detect the effects of LDHA knockdown or overexpression on the invasion of TMEM105-overexpressing MCF-7 cells (**C**) and TMEM105-knockdown MDA-MB-231 cells (**D**). **E** Representative HE images of the livers from nude mice without or with BCLM. Scale bar, 50 μm. **F**, **G** The BCLM in the mice was determined by qRT-PCR assays. **H** Western blot analysis of LDHA expression in liver metastatic lesions from nude mice injected with engineered breast cancer cells. **I**, **J** Changes of the overall survival in nude mice injected with engineered MCF-7 (**I**) and MDA-MB-231 (**J**) cells (*n* = 8 for each group). **p < 0.01.

We further examined the role of LDHA in TMEM105-mediated metastasis of breast cancer cells. The increase or decrease in wound healing after TMEM105 overexpression or knockdown was significantly reversed by knockdown or overexpression of LDHA, respectively (Fig. 4A, B). Moreover, similar results were obtained in the cell invasion assay (Fig. 4C, D). It suggested that TMEM105 enhanced the migration and invasion of breast cancer cells in an LDHA-dependent manner.

Furthermore, we injected engineered MCF-7 and MDA-MB-231 cells into the spleen of nude mice (Fig. 4E). The mice were sacrificed and the livers were collected for detection of human genomic DNA by qRT-PCR in accordance with previous studies [20]. It showed that overexpression or downregulation of TMEM105 promoted or suppressed the human genomic DNA and LDHA expression in the livers, and these effects were abrogated after the knockdown or overexpression of LDHA (Fig. 4F–H). We also found that OS was greatly decreased or increased in TMEM105-overexpressing or knockdown mice, and these effects were reversed after LDHA knockdown or overexpression, respectively (Fig. 4I, J). Thus, these results indicated that TMEM105 contributed to BCLM in vivo via an LDHA-dependent manner.

### TMEM105 enhances LDHA expression through competitive interaction with miR-1208

**Fig. 5:**
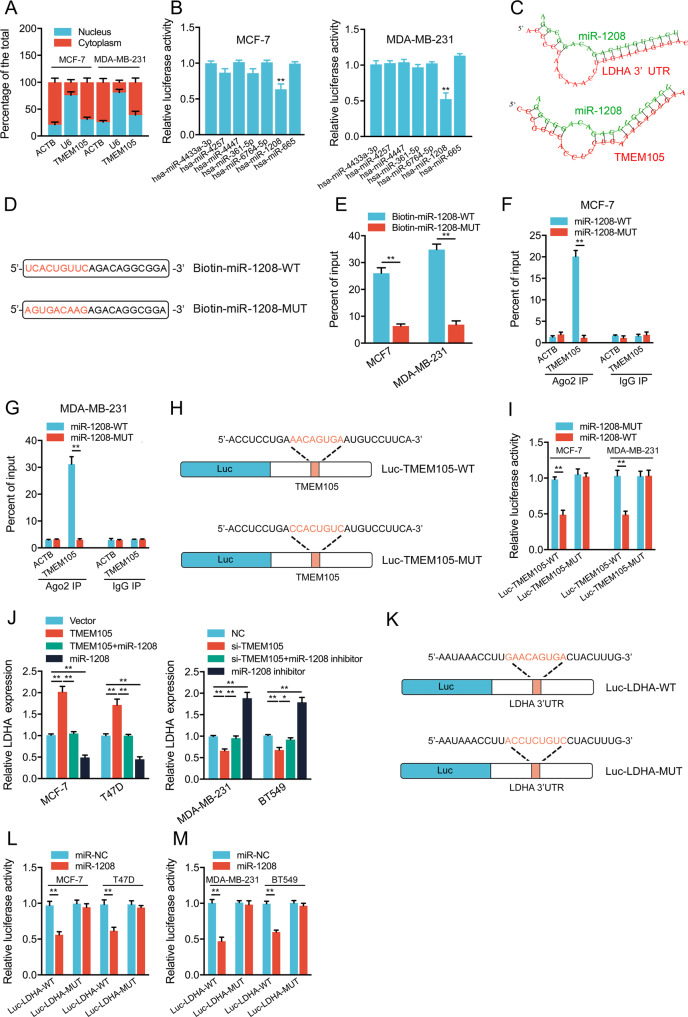
TMEM105 sponges miR-1208 to upregulate LDHA expression in breast cancer cells. **A** The subcellular localization analysis of TMEM105 in breast cancer cells was measured by qRT-PCR. ACTB was the positive control for the cytoplasm, and U6 was the positive control for the nucleus. **B** Overexpression of the potential candidate miRNAs on the luciferase activity of TMEM105**-**WT in MCF-7 and MDA-MB-231 cells. **C** The binding sites in miR-1208, TMEM105, and the LDHA 3′UTR were predicted using the RNAhybrid program. **D** The schematic images of miR-1208-WT and miR-1208-MUT. **E** MCF-7 and MDA-MB-231 cell lysates were incubated with biotin-labeled miR-1208-WT or miR-1208-MUT. The expression of TMEM105 in pull-down products was analyzed by qRT-PCR. **F**, **G** Ago2-RIP followed by qRT-PCR to evaluate TMEM105 expression after transfection with miR-1208-WT or miR-1208-MUT in MCF-7 (**F**) and MDA-MB-231 (**G**) cells. **H** The schematic images of reporter genes contained TMEM105-WT or TMEM105-MUT without the miR-1208 binding site. **I** Effects of miR-1208-WT or miR-1208-MUT on the luciferase activity with the TMEM105-WT or TMEM105-MUT in MCF-7 and MDA-MB-231 cells. **J** Effects of miR-1208 mimics or inhibitors on LDHA levels in the TMEM105-overexpressing or knockdown cells. **K** The schematic images of reporter genes contained LDHA 3′ UTR wild or the mutant type without the miR-1208 binding site. (**L**, **M**) Effects of miR-1208 on the luciferase activity with the LDHA-WT or LDHA-MUT in breast cancer cells. *p < 0.05, **p < 0.01.

To further explore the regulatory mechanism of TMEM105, we used β-actin as a cytoplasmic control and U6 as a nuclear control to observe the subcellular localization of TMEM105. It showed that the expression of TMEM105 was significantly higher in the cytoplasm than in the nucleus (Fig. 5A). Since cytoplasmic lncRNAs can act as sponges of miRNAs and rescue expression of the miRNA targets [21, 22], whether TMEM105 regulates LDHA mRNA expression by acting as a miRNA sponge is not clear. We predicted a total of seven candidate miRNAs targeting the 3′ UTR of LDHA mRNA using the software TargetScan, miRDB, and miRanda. To evaluate whether these predicted candidate miRNAs are interacting with TMEM105, we constructed a luciferase reporter vector containing the wild type of TMEM105 (Luc-TMEM105-WT) and cotransfected it with miRNA mimics into the breast cancer cells. It showed that miR-1208 had the most effective inhibitory effect on luciferase activity in MCF-7 and MDA-MB-231 cells (Fig. 5B). Further it was shown that TMEM105 and the 3′ UTR of LDHA mRNA might share the same miR-1208 binding sequence using RNAhybrid simulations (Fig. 5C). Then the results showed that TMEM105 was more abundant in the precipitates of wild-type biotin-coupled miR-1208 than in the mutant precipitates (Fig. 5D, E). Moreover, TMEM105 was enriched in wild-type miR-1208-transfected cells after Ago2 immunoprecipitation than in mutant miR-1208-transfected cells, suggesting that Ago2 is essential in the interaction between TMEM105 and miR-1208 (Fig. 5F, G). Furthermore, we constructed the TMEM105 mutant fragment and inserted it downstream of the luciferase reporter gene (Luc-TMEM105-MUT) (Fig. 5H). It showed that wild-type miR-1208 dramatically reduced the luciferase activity compared to the mutant group, while transfection with Luc-TMEM105-MUT could reverse this effect (Fig. 5I).

In addition, transfection with miR-1208 mimics or inhibitors significantly attenuated the effects of TMEM105 increase or decrease on LDHA expression (Fig. 5J). Moreover, the luciferase reporter vectors containing the wild-type 3′ UTR of LDHA mRNA (Luc-LDHA-WT) or mutant 3′ UTR sequence (Luc-LDHA-MUT) were constructed (Fig. 5K), and it revealed that luciferase activity was dramatically decreased in miR-1208 mimics transfected, whereas the luciferase activity of Luc-LDHA-MUT-transfected cells was not affected (Fig. 5L, M). These results suggested that TMEM105 enhanced LDHA expression by sponging miR-1208 in breast cancer cells.

### TMEM105 facilitates glycolysis in breast cancer cells by sponging miR-1208

**Fig. 6:**
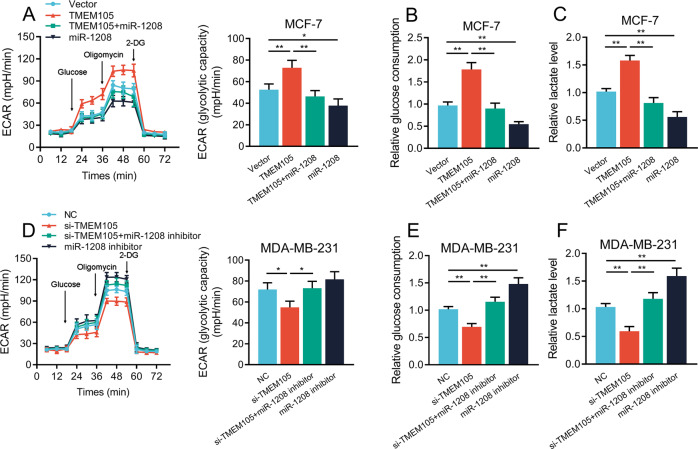
TMEM105 sponges miR-1208 to facilitate breast cancer cell glycolysis. ECAR level (**A**), glucose consumption (**B**), and lactate production (**C**) were determined in TMEM105-overexpressing MCF-7 cells transfected with miR-1208 mimics. ECAR level (**D**), glucose consumption (**E**), and lactate production (**F**) were determined in TMEM105-knockdown MDA-MB-231 cells transfected with miR-1208 inhibitors. 2-DG 2-deoxyglucose. *p < 0.05, **p < 0.01.

Based on these findings, we addressed whether TMEM105 promoted glycolysis in breast cancer cells by sponging miR-1208. The miR-1208 mimics or inhibitors were transfected into TMEM105-overexpressing MCF-7 cells or TMEM105-knockdown MDA-MB-231 cells, respectively. It showed that miR-1208 mimic or miR-1208 inhibitor transfected into TMEM105-overexpressing or knockdown cells resulted in significant inhibition or promotion of ECAR, glucose consumption, and lactate production (Fig. 6A–F). These findings suggested that TMEM105 regulated cellular glycolysis by sponging miR-1208.

### TMEM105 functions as ceRNA to enhance breast cancer cell invasion and BCLM

**Fig. 7:**
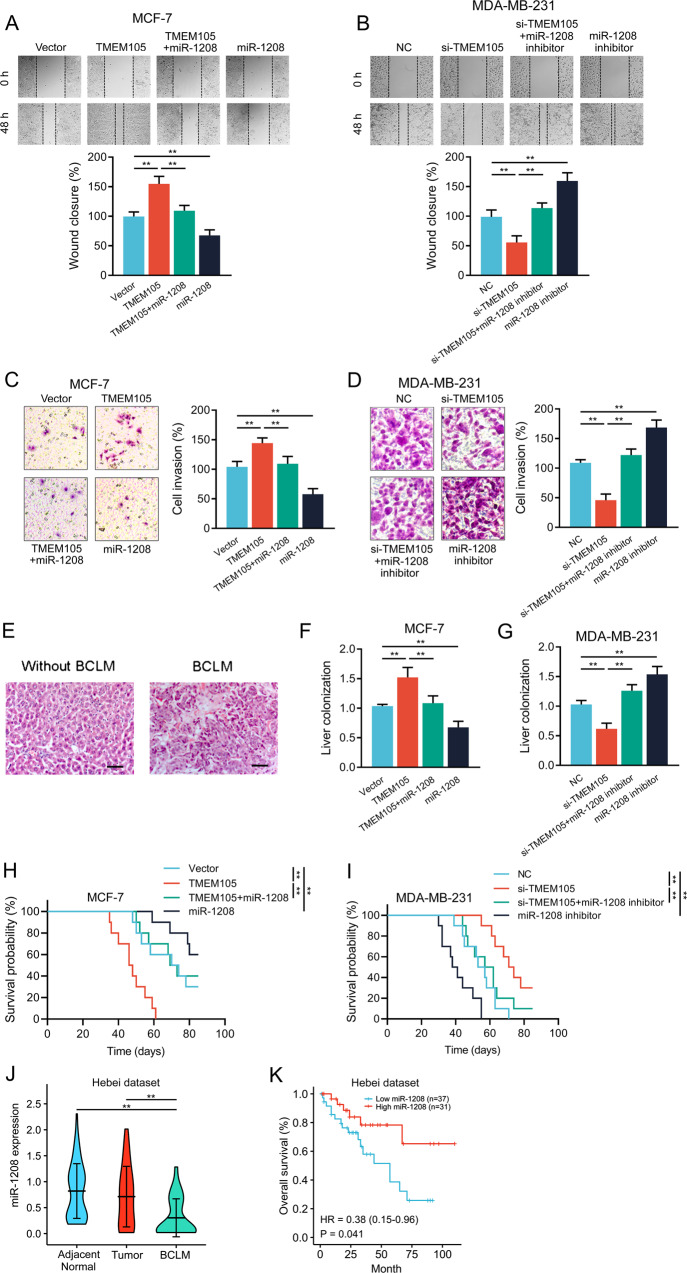
TMEM105 sponges miR-1208 to enhance breast cancer cell invasion and BCLM. **A**, **B** Wound healing assay was performed to detect the effects of miR-1208 mimics or inhibitors on the migration of TMEM105-overexpressing MCF-7 cells (**A**) and TMEM105-knockdown MDA-MB-231 cells (**B**). **C**, **D** Transwell matrigel assay was performed to detect the effects of miR-1208 mimics or inhibitors on the invasion of TMEM105-overexpressing MCF-7 cells (**C**) and TMEM105-knockdown MDA-MB-231 cells (**D**). **E** Representative HE images of the livers from nude mice without or with BCLM. Scale bars, 50 μm. **F**, **G** The BCLM in the mice was determined by qRT-PCR assays. **H**, **I** Changes of the overall survival in nude mice injected with engineered MCF-7 (**H**) and MDA-MB-231 (**I**) cells (*n* = 8 for each group). **J** miR-1208 expression in adjacent normal tissues, breast cancer, and paired BCLM in the Hebei dataset (*n* = 68). **K** Kaplan–Meier analysis for OS according to TMEM105 expression in the Hebei dataset. **p < 0.01.

We further transfected miR-1208 mimics into TMEM105-overexpressing breast cancer cells or transfected miR-1208 inhibitors into TMEM105-knockdown breast cancer cells, which attenuated the effects of TMEM105 overexpression or knockdown on wound healing, respectively (Fig. 7A, B). Moreover, similar results were observed in transwell cell invasion assays (Fig. 7C, D). Thus, these data suggested that TMEM105 regulated migration and invasion of breast cancer cells in vitro by sponging miR-1208.

To further confirm the epigenetic effect of TMEM105 on miR-1208 in vivo, we injected breast cancer cells into the spleen of nude mice. The results revealed that the injection of TMEM105-overexpressing or knockdown breast cancer cells promoted or suppressed the human genomic DNA in the livers, and these effects were reversed after cotransfection with miR-1208 mimics or inhibitors (Fig. 7E–G). In addition, breast cancer cells transfected with miR-1208 mimics or miR-1208 inhibitors were able to completely reverse the effect of TMEM105 overexpression or knockdown on OS time in the mice (Fig. 7H, I). Notably, the analysis of clinical data from our institution indicated that miR-1208 expression was lower in cancer tissues with BCLM than in adjacent normal and cancer tissues without BCLM (Fig. 7J). Moreover, breast cancer patients with low miR-1208 expression had a shorter OS time by survival analysis (Fig. 7K).

### Lactate induces TMEM105 expression by the SHH-MAZ signaling pathway in a positive feedback loop

**Fig. 8:**
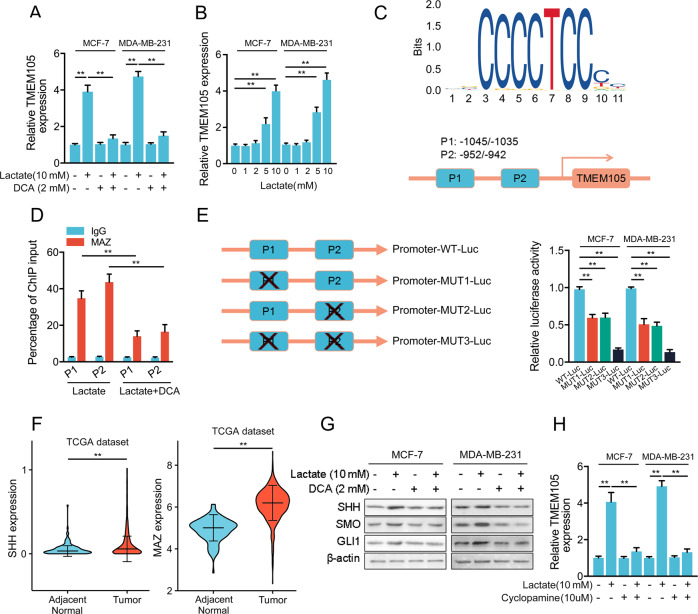
Lactate promotes TMEM105 expression by SHH-MAZ signaling pathway. **A** The lactate secretion inhibitor DCA (2 mM) downregulated TMEM105 expression in MCF-7 and MDA-MB-231 cells treated with lactate (10 mM) for 24 h. **B** TMEM105 expression was determined by qRT-PCR in MCF-7 and MDA-MB-231 cells treated with different concentrations of lactate for 24 h. **C** The schematic images of the MAZ binding motif and the potential binding sites of MAZ in the TMEM105 promoter. **D** ChIP assay was used to detect the roles of MAZ binding sites on the TMEM105 promoter region. **E** TMEM105 reporter vectors with truncated mutants in the promoter region at site P1, site P2, or both sites were transfected into MCF-7 or MDA-MB-231 cells treated with lactate (10 mM), and luciferase activity was detected. **F** SHH and MAZ expression in adjacent normal and breast cancer tissues in the TCGA dataset. **G** Expression of SHH, SMO, and GLI1 was examined in breast cancer cells using Western blot. **H** Effects of cyclopamine (10 μM) on TMEM105 expression in breast cancer cells treated with lactate (10 mM). **p < 0.01.

Glucose is mainly processed into lactate during glycolysis. Importantly, inhibition of cellular lactate production abrogated the upregulation of TMEM105 (Fig. 8A), indicating that the glycolytic lactate production may in turn regulate the expression of TMEM105. Furthermore, we observed that lactate level was associated with the upregulation of TMEM105, and 5 mM of lactate was enough to dramatically induce the TMEM105 expression in breast cancer cells (Fig. 8B). To elucidate the mechanism by which lactate promotes TMEM105 transcription, we screened TMEM105 promoter sequence from −2000 bp upstream to +100 bp downstream of the transcription start site using JASPAR, and predicted two potential MAZ binding sites in TMEM105 promoter (Fig. 8C). ChIP assays were performed to determine whether MAZ binds directly to TMEM105 promoter. It showed that lactate significantly promoted the enrichment of MAZ at both position 1 (−1045 bp to −1035 bp) and position 2 (−952 bp to −942 bp) in the TMEM105 promoter (Fig. 8D). Additionally, we prepared three truncated mutants of TMEM105 promoter, with truncated mutations at position 1 (Promoter-MUT1-Luc), position 2 (Promoter-MUT2-Luc) or both positions (Promoter-MUT3-Luc), and cloned them into pGL3 vector and transfected them into breast cancer cells. The luciferase reporter assay indicated that deletion of either MAZ binding site alone dramatically decreased promoter activity, and simultaneous deletion of both sites further reduced the promoter activity, suggesting that the MAZ binding site may be essential for TMEM105 transcription after lactate treatment (Fig. 8E).

Previous studies have shown that MAZ is a transcription factor downstream of the SHH signaling pathway [23]. Analysis of TCGA data showed that SHH and MAZ were significantly overexpressed in breast cancer tissues (Fig. 8F). We also observed that the accumulation of lactate significantly increased SHH, SMO, and GLI1 expression of the SHH signaling pathway in breast cancer cells (Fig. 8G). Furthermore, after lactate treatment, TMEM105 expression was abrogated in the cells with SHH inhibitor cyclopamine (Fig. 8H). Thus, these data suggested that lactate activated the SHH-MAZ signaling pathway, which promoted MAZ binding to the TMEM105 promoter, further upregulating TMEM105 expression in breast cancer cells, constituting a positive feedback loop to induce sustained glycolysis.

## Discussion

BCLM is one of the leading reasons of death in breast cancer patients [4, 6, 24]. Glycolysis has been shown to produce large amounts of lactate, which forms the acidic microenvironment outside the cells and induces the degradation of the extracellular matrix, thus promoting the metastasis of cancer cells [25–27]. Here, TMEM105 was significantly upregulated in breast cancer tissues, facilitating glycolysis and promoting BCLM. In addition, lactate generated by glycolysis also enhanced TMEM105 expression through the SHH-MAZ pathway in turn, forming an important regulatory feedback loop between TMEM105 expression and glycolysis. This study indicated that TMEM105 was involved in the metabolic conversion and reprogramming of glucose, which could be a novel therapeutic target for patients with BCLM.

Glycolysis is not only the primary source of energy production in cancer cells but also influences gene transcription and regulates multiple signaling pathways [8, 26, 28]. The glycolytic reprogramming provides a selective growth advantage for cancer cells, but it is not clear about the regulatory mechanisms of glycolytic metabolic conversion in the cells. When we investigated the mechanisms by which TMEM105 could mediate glycolysis and regulate BCLM in breast cancer cells, we found the involvement of LDHA, which was a critical component of the glycolytic pathway and played a key role in the proliferation, migration, and chemoresistance of various cancer cells [29–31]. LDHA is implicated in the regulation of glycolysis and is known to be regulated by PI3K signaling and hypoxia-inducible factor 1 (HIF-1α) expression [32]. It has been shown that the expression of LDHA is notably elevated in breast cancer [33, 34]. The aberrant activation of LDHA by TMEM105 might be the result of the metabolic adaptation for the survival of breast cancer cells in the cancer microenvironment. It can be speculated that TMEM105 is a potential key molecule implicated in reprogramming metabolic conversions during glucose deficiency.

There is increasing evidence showing the involvement of lncRNAs in the biological behaviors of cancer cell proliferation, apoptosis, and metastasis [13]. The miRNAs are widely dysregulated in several cancers and have a key role in the regulation of cancer development [35, 36]. Moreover, it has been shown that miRNAs function as important regulators in different physiological and pathological processes by targeting the mRNAs [37]. In the cytoplasm, lncRNAs could act as sponges for miRNAs and prevent miRNA-induced gene silencing [21, 22]. It has been reported that miR-1208 participates in cancer progression and drug susceptibility by modifying a series of targeted genes [38, 39]. Here, we found that TMEM105 was enriched in the cytoplasm. As a sponge of miR-1208, TMEM105 upregulated LDHA expression by competitively interacting with miR-1208 in breast cancer cells thereby promoting glycolysis and BCLM. Thus, the evaluation of the combination of TMEM105, miR-1208, and LDHA is valuable for predicting the prognosis of BCLM patients. In addition, the glycolytic production of lactate induced TMEM105 expression through the SHH-MAZ pathway. Recently, lactate has been proven to affect tumor invasion by acidification of the microenvironment and to be involved in regulating cell motility functions [40]. It has been proved that lactate can act as a signaling molecule involved in cellular messaging, and studies have shown that lactate is involved in pituitary adenoma cell invasion via the mTORC2 and ERK signaling pathways [41]. Previous reports provided evidence that SHH signaling was involved in drug resistance and directly influenced metastasis in breast cancer [42]. Our study identified a new role for the SHH signaling pathway in modulating lncRNA expression and BCLM, which suggested a novel mechanism involving this pathway and glycolysis. An understanding of the roles of glucose metabolism in cellular regulation will be helpful in identifying the therapeutic targets and the development of oncologic diagnostic reagents.

## Conclusions

In summary, our study provided a novel mechanism of BCLM development by examining the biological cross-talk between TMEM105, LDHA, miR-1208, lactate, and SHH-MAZ signaling pathway. Our findings may contribute to effective strategies for the prevention and treatment of metastatic breast cancer.

## Materials and methods

### Cell culture and transfection

Human breast cancer cell lines (MCF-7, T47D, MDA-MB-231, and BT549) and normal breast epithelial cell MCF10A were purchased from the Chinese Academy of Sciences Cell Bank (Shanghai, China) or the American Type Culture Collection (Manassas, USA). The cell lines were cultured in Dulbecco’s modified Eagle’s medium (Gibco, USA) containing 10% fetal bovine serum (Invitrogen, USA) and 1% penicillin-streptomycin at 37 °C in a humidified 5% CO_2_ atmosphere. Transfection of siRNAs was performed with Lipofectamine RNAiMAX (Invitrogen, USA) according to the manufacturer’s instructions. The miRNA mimics and specific miRNA inhibitor were purchased from Genepharma (Shanghai, China). Lentiviral vectors for overexpression of TMEM105 and LDHA were constructed by RiboBio (Guangzhou, China).

### Patients and clinical samples

The adjacent normal tissues, breast cancer, and paired BCLM used in this study were surgical specimens from 68 patients who underwent tumor resection and did not receive preoperative therapy. All the samples were separated into two parts, one part was stored at −80 °C, and the other part was formalin-fixed. The study protocol was approved by the ethics committee of the Second Hospital of Hebei Medical University (Hebei, China), and informed consent was obtained from the patients before sample collection.

### Bioinformatics analysis

The data on primary breast cancer and BCLM were downloaded from the Gene Expression Omnibus (GSE110590). The dataset GSE110590 consisted of 12 primary breast cancer and 14 BCLM patient samples. Other breast cancer data and the correlated clinic data were obtained from the TCGA dataset including 1091 breast cancer samples and 113 adjacent normal breast samples. To understand the biological pathways involved in breast cancer via TMEM105, GSEA was performed using the R package clusterProfiler. FDR < 0.25 and *p* < 0.05 were considered statistically significant.

### RNA extraction and quantitative real-time PCR (qRT-PCR)

Total RNA was extracted with TRIzol reagent (Invitrogen, USA) and reverse transcribed into cDNA. The qRT-PCR was performed with the SYBR PrimeScript RT-PCR kit (Takara) according to the manufacturer’s instructions. The amplified transcript levels were normalized to appropriate internal control (β-Actin or U6). The primers were provided by Shenggong Company (Shanghai, China). The primer sequences for the qRT-PCR assay are listed in Supplementary Table S2.

### Measurement of extracellular acidification rate (ECAR)

The ECAR was measured with Seahorse Extracellular Flux Analyzer XF96 (Seahorse Bioscience, USA). Briefly, 10^5^ cells were seeded and exposed to 10 mM glucose, followed by 1 µM oligomycin, and 100 mM 2-deoxyglucose (2-DG). Three replicate measurements of ECAR were performed for each group.

### Glucose consumption and lactate production assays

Glucose consumption was determined using the Glucose Colorimetric/Fluorometric Assay Kit (Biovision, USA). The level of lactate was determined using the L-lactate Assay Kit (Abcam, USA) according to the manufacturer’s protocol.

### Wound healing and invasion assay

For the wound healing assays, cells were treated and cultured in six-well plates. The wound was made using a sterile pipette tip and the images were randomly acquired at 0 and 48 h under the microscope.

Cell invasion assays were performed with the Transwell chambers (8 μm pores, Corning, USA) that were coated with Matrigel (BD Biosciences, USA). Cells were seeded into the top chamber, and 10% FBS in medium was added to the bottom chamber. After 48 h, the cells that invaded through the membrane were fixed, stained with crystal violet, and counted in three fields under an inverted microscope.

### Western blot analysis

For Western blot analysis, the cell lysates were prepared as previously described [17]. After determination of the protein concentrations, proteins were separated by SDS-PAGE and transferred onto PVDF membranes, then the membranes were exposed to the following antibodies: PGK1 (Abcam), PKM2 (Proteintech), LDHA (Proteintech), SHH (Abcam), SMO (Abcam), GLI1 (Proteintech), and β-actin (Proteintech). Protein bands were visualized using corresponding secondary antibodies and detected using the ECL detection system.

### Immunohistochemistry analysis

Immunohistochemical (IHC) analysis of protein expression was performed with formalin-fixed surgical sections obtained from the patients. Sections were incubated with LDHA antibody (1:100, 19987-1-AP, Proteintech) at 4 °C overnight. The diaminobenzidine was used as the substrate for visualization, and counterstaining was performed with hematoxylin. IHC staining scores were used to determine the high or low expression of target proteins. The percentage of positive cells was classified as 1, <25%; 2, 25–50%; 3, 50–75%; 4, >75%. Moreover, the intensity of IHC staining was classified as 0 = negative; 1 = weak staining; 2 = moderate staining; 3 = strong staining. IHC staining score = positive percentage × intensity.

### In situ hybridization (ISH)

ISH was performed to examine the presence of TMEM105 with digoxigenin-labeled probes synthesized by Exiqon (Shanghai, China). ISH Kit (Boster Bio-Engineering Company, China) was used to conduct the assay. In addition, the ISH score was quantified by the following criteria: 0 = negative; 1 = weak staining; 2 = moderate staining; 3 = strong staining.

### RNA pull-down assay

Biotin-labeled RNAs were transcribed with Biotin RNA Labeling Mix (Roche) and T7 RNA polymerase (Invitrogen, USA), treated with RNase-free DNase I (Invitrogen, USA), and purified with the RNeasy Mini Kit (Qiagen, Germany). Cell lysates were incubated with biotin-labeled RNAs overnight. The proteins associated with biotin-labeled RNAs were pulled down with streptavidin magnetic beads (Sigma, USA) and subjected to immunoblot analysis.

### RNA immunoprecipitation (RIP)

The RIP assay was performed with the Magna RIP RNA Binding Protein Immunoprecipitation Kit (Millipore, USA) according to the manufacturer’s instructions. Briefly, cells were lysed by RIP lysis buffer containing protease inhibitor cocktail and RNase inhibitors (Invitrogen, USA). The cell extracts were incubated with magnetic beads conjugated to anti-Ago2 antibody (Cell signaling) or the negative control IgG. Then the protein-RNA complexes were digested with Proteinase K buffer, and the co-immunoprecipitated RNA was then eluted and processed for qRT-PCR analysis.

### Luciferase reporter assay

The wild-type or mutated fragments of the TMEM105 and the 3′-UTR of LDHA with or without the miR-1208 putative binding sites were synthesized by GenePharma (Shanghai, China), and then they were inserted into pGL3 firefly luciferase vector. The miRNA mimics, miRNA inhibitors, or negative controls were cotransfected with luciferase reporter into the cells using Lipofectamine 2000 following the manufacturer’s instructions (Invitrogen, USA). Luciferase activities were determined 48 h later using the Dual Luciferase Assay System (Promega), and the Renilla fluorescence was used as the internal control to calculate the relative luciferase activity.

### Chromatin immunoprecipitation (ChIP) assay

The ChIP experiments were performed using EZ-ChIP Kit (Millipore, USA). Briefly, 1 × 10^6^ cells were fixed in 1% formaldehyde at room temperature. After cell lysis, the isolated nuclei were subjected to sonication and sheared to lengths between 100 and 200 bp. Antibodies against MAZ (Abcam) or IgG control (Cell Signaling) were added to chromatin samples, followed by overnight incubation with rotation. Immune complexes were captured using magnetic protein A/G beads. Purified DNAs were subjected to qRT-PCR. The primer sequences used in the ChIP assay are listed in Supplementary Table S2.

### Metastasis mouse models

The experimental liver-metastasis animal model was established by splenic injection of tumor cells [28, 43]. The protocols of the experiment were approved by the Institutional Animal Care and Use Committee of the Second Hospital of Hebei Medical University (Hebei, China). The female BALB/c-nude mice (5–6 weeks old) were anesthetized and PBS containing 5 × 10^5^ MCF-7 or MDA-MB-231 cells (10 μl) was injected into the spleen using a 30-gauge needle. Six mice were euthanized 5 weeks after the implantation, at which time the livers were collected and perfused with PBS and subjected to qRT-PCR with primers for the human HK2 gene and mouse and human 18S rRNA [20]. The remaining mice were monitored until week 12 for survival analysis.

### Statistical analysis

Statistical validation between two independent groups was done with a two-tailed Student’s *t*-test. Comparison of multiple groups was made using a one- or two-way ANOVA test. The overall survival (OS) curve was calculated using the Kaplan–Meier method and differences were assessed using the log-rank test. Spearman’s correlation test was used to test the relationship between numerical variables. Univariate and multivariate Cox regression analyses were performed to explore the effect of selected variables on OS. Data were expressed as mean ± SD. Differences with *p* < 0.05 were considered statistically significant.

## Supplementary information

SUPPLEMENTAL MATERIAL

Western blots

aj-checklist

## Data Availability

All data generated or analyzed during this study are included in this published article.
