# Supplementary material for: Glycolysis-related lncRNA TMEM105 upregulates LDHA to facilitate breast cancer liver metastasis via sponging miR-1208
Source: Cell Death Dis. 2023 Feb 3;14(2):80. doi: 10.1038/s41419-023-05628-z (PMC9898275; doi:10.1038/s41419-023-05628-z)
Supplement: Supplementary file 1 — SUPPLEMENTAL MATERIAL [file 41419_2023_5628_MOESM1_ESM.doc]

**Figure S1.**

**
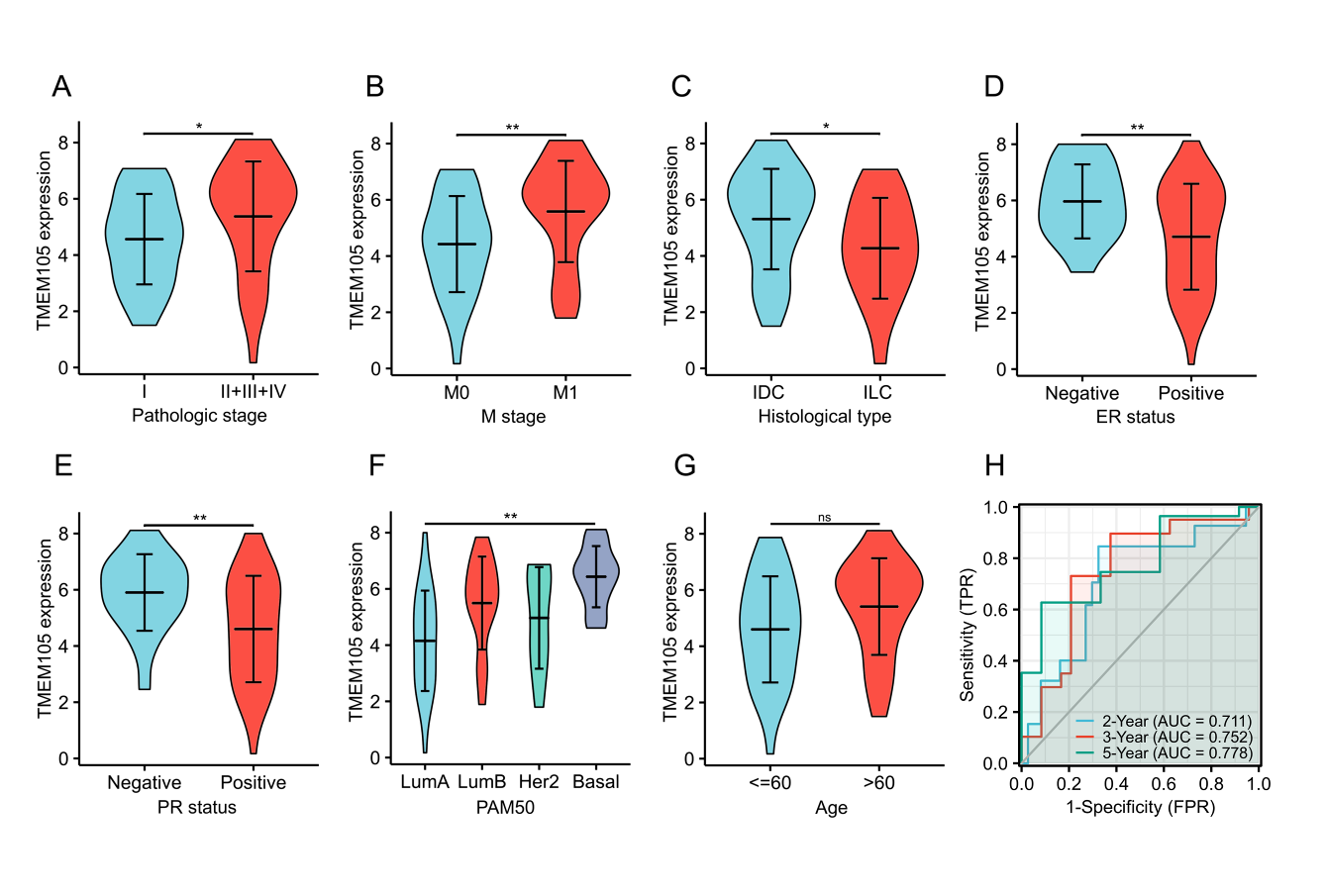
Figure S1.** Clinical relevance of TMEM105 in breast cancer in the Hebei dataset. Correlations of TMEM105 expression with pathological grade (**A**), distant metastasis (**B**), histological type (**C**), ER status (**D**), PR status (**E**), PAM50 subtype (**F**) and age (**G**) in the breast cancer patients. (**h**) Time-dependent ROC curve analysis was constructed in Hebei dataset. *p< 0.05, **p< 0.01.

### Figure S2.

###
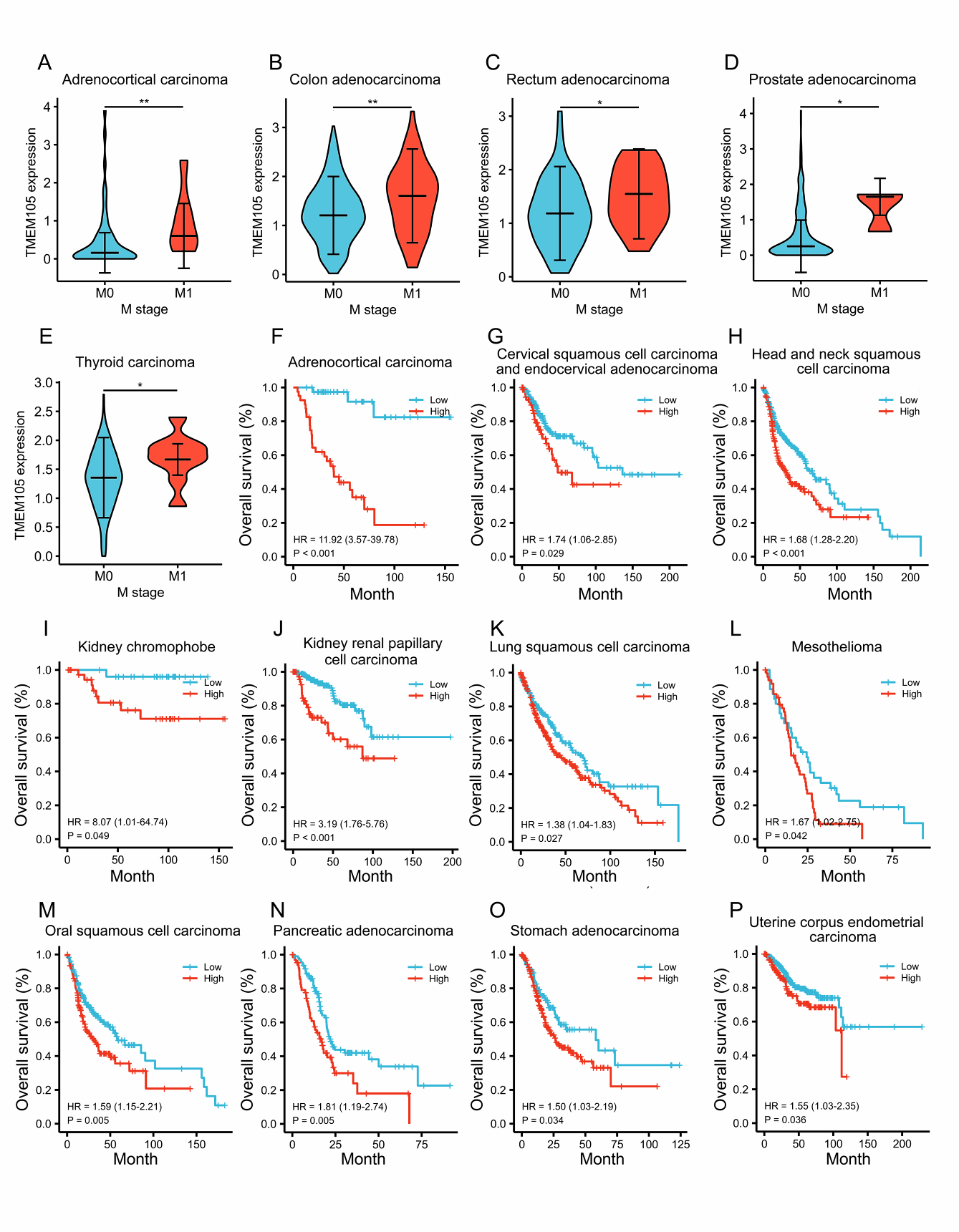


**Figure S2.** TMEM105 expression of different cancer types was analyzed in the TCGA dataset. (**a-e**) Analysis of TCGA dataset revealed that TMEM105 correlated with metastasis in different cancers. (**f-p**) Kaplan-Meier survival analysis was performed to compare the OS of the patients with different cancers according to TMEM105 expression in the TCGA dataset. The median TMEM105 expression was used as the cutoff value. *p < 0.05, **p < 0.01.

**Table S1.** Univariate and multivariate Cox regression analyses of overall survival in breast cancer patients in the Hebei dataset.

| Characteristics | Univariate analysis | |  | Multivariate analysis | |
| --- | --- | --- | --- | --- | --- |
|  | Hazard ratio (95% CI) | P value |  | Hazard ratio (95% CI) | P value |
| Pathologic stage (II+III+IV versus I) | 11.525 (3.066-43.314) | <0.001 |  | 13.241 (1.893-92.632) | 0.009 |
| Tumor size (T3+T4 versus T1+T2) | 1.054 (0.391-2.841) | 0.918 |  |  |  |
| Lymph node metastasis (N2-3 versus N0-1) | 1.273 (0.362-4.478) | 0.706 |  |  |  |
| M stage (M1 versus M0) | 7.617 (2.401-24.164) | <0.001 |  | 0.466 (0.074-2.941) | 0.416 |
| Histological type (ILC versus IDC) | 0.616 (0.242-1.568) | 0.310 |  |  |  |
| ER status (Positive versus Negative) | 0.848 (0.334-2.153) | 0.729 |  |  |  |
| PR status (Positive versus Negative) | 0.858 (0.351-2.102) | 0.738 |  |  |  |
| Age (>60 versus ≤60) | 2.450 (1.040-5.770) | 0.040 |  | 2.146 (0.777-5.931) | 0.141 |
| PAM50 (LumB versus LumA) | 2.144 (0.756-6.078) | 0.151 |  |  |  |
| PAM50 (Her2 versus LumA) | 2.208 (0.439-11.118) | 0.337 |  |  |  |
| PAM50 (Basal versus LumA) | 2.482 (0.866-7.110) | 0.090 |  |  |  |
| TMEM105 expression (high versus low) | 0.278 (0.114-0.679) | 0.005 |  | 0.275 (0.090-0.843) | 0.024 |

**Table S2.** Primer and siRNA sequence used in this study.

| **Type** | **Gene** |  | **Sequence** |
| --- | --- | --- | --- |
| **Primer** | GLUT1 | Forward | ATTGGCTCCGGTATCGTCAAC |
|  |  | Reverse | GCTCAGATAGGACATCCAGGGTA |
|  | HK1 | Forward | GCTCTCCGATGAAACTCTCATAG |
|  |  | Reverse | GGACCTTACGAATGTTGGCAA |
|  | HK2 | Forward | TGCCACCAGACTAAACTAGACG |
|  |  | Reverse | CCCGTGCCCACAATGAGAC |
|  | PFK1 | Forward | AGCTGCCTACAACCTGGTGA |
|  |  | Reverse | TCCACTCAGAACGGAAGGTGT |
|  | PGK1 | Forward | GAACAAGGTTAAAGCCGAGCC |
|  |  | Reverse | GTGGCAGATTGACTCCTACCA |
|  | PKM2 | Forward | ATGGCTGACACATTCCTGGAGC |
|  |  | Reverse | CCTTCAACGTCTCCACTGATCG |
|  | LDHA | Forward | TTGACCTACGTGGCTTGGAAG |
|  |  | Reverse | GGTAACGGAATCGGGCTGAAT |
|  | LDHB | Forward | TGGTATGGCGTGTGCTATCAG |
|  |  | Reverse | TTGGCGGTCACAGAATAATCTTT |
|  | TMEM105 | Forward | ATGAAGATAAGAAGGCGA |
|  |  | Reverse | GGTGAAAAACACGATGAG |
|  | β-Actin | Forward | GTCATTCCAAATATGAGATGCGT |
|  |  | Reverse | GCATTACATAATTTACACGAAAGCA |
|  | U6 | Forward | CTCGCTTCGGCAGCACA |
|  |  | Reverse | AACGCTTCACGAATTTGCGT |
|  | 18S rRNA | Forward | CGGCGACGACCCATTCGAAC |
|  |  | Reverse | GAATCGAACCCTGATTCCCCGTC |
|  | hsa-miR-4433a-3p | RT-hsa-miR-4433a-3p | CCTGTTGTCTCCAGCCACAAAAGAGCACAATATTTCAGGAGACAACAGGATGTCCC |
|  |  | Forward | CGGGCACAGGAGTGGGGGT |
|  |  | Reverse | CAGCCACAAAAGAGCACAAT |
|  | hsa-miR-4257 | RT-hsa-miR-4257 | CCTGTTGTCTCCAGCCACAAAAGAGCACAATATTTCAGGAGACAACAGGCTCAGTC |
|  |  | Forward | CGGGCCCAGAGGTGGG |
|  |  | Reverse | CAGCCACAAAAGAGCACAAT |
|  | hsa-miR-4447 | RT-hsa-miR-4447 | CCTGTTGTCTCCAGCCACAAAAGAGCACAATATTTCAGGAGACAACAGGAAACAAC |
|  |  | Forward | CGGGCGGTGGGGGCT |
|  |  | Reverse | CAGCCACAAAAGAGCACAAT |
|  | hsa-miR-361-5p | RT-hsa-miR-361-5p | CCTGTTGTCTCCAGCCACAAAAGAGCACAATATTTCAGGAGACAACAGGGTACCCC |
|  |  | Forward | CGGGCTTATCAGAATCTCCA |
|  |  | Reverse | CAGCCACAAAAGAGCACAAT |
|  | hsa-miR-6764-5p | RT-hsa-miR-6764-5p | CCTGTTGTCTCCAGCCACAAAAGAGCACAATATTTCAGGAGACAACAGGCAACTCT |
|  |  | Forward | CGGGCTCCCAGGGTCTGGTC |
|  |  | Reverse | CAGCCACAAAAGAGCACAAT |
|  | hsa-miR-1208 | RT-hsa-miR-1208 | CCTGTTGTCTCCAGCCACAAAAGAGCACAATATTTCAGGAGACAACAGGTCCGCCT |
|  |  | Forward | CGGGCTCACTGTTCAGAC |
|  |  | Reverse | CAGCCACAAAAGAGCACAAT |
|  | hsa-miR-665 | RT-hsa-miR-665 | CCTGTTGTCTCCAGCCACAAAAGAGCACAATATTTCAGGAGACAACAGGAGGGGCC |
|  |  | Forward | CGGGCACCAGGAGGCTGA |
|  |  | Reverse | CAGCCACAAAAGAGCACAAT |
| **ChIP-qPCR primers** | TMEM105 promoter ChIP-P1 | Forward | CTCTGAGAGGTTGCGGCTG |
|  |  | Reverse | TGTGGTGCAGAGCAACTGTG |
|  | TMEM105 promoter ChIP-P2 | Forward | GCACCACACAGCTCCGCT |
|  |  | Reverse | CATGCCTTATCCTCAGCAGCTC |
| **siRNA** | si-TMEM105 | Sense | CAAGACUCUCUCAAAUCAATT |
|  |  | Antisense | UUGAUUUGAGAGAGUCUUGTT |
|  | si-LDHA | Sense | GGCAAAGACUAUAAUGUAATT |
|  |  | Antisense | UUACAUUAUAGUCUUUGCCTT |
