# Supplementary material for: Glycolysis-related lncRNA TMEM105 upregulates LDHA to facilitate breast cancer liver metastasis via sponging miR-1208
Source: Cell Death Dis. 2023 Feb 3;14(2):80. doi: 10.1038/s41419-023-05628-z (PMC9898275; doi:10.1038/s41419-023-05628-z)
Supplement: Supplementary file 2 [file 41419_2023_5628_MOESM2_ESM.doc]

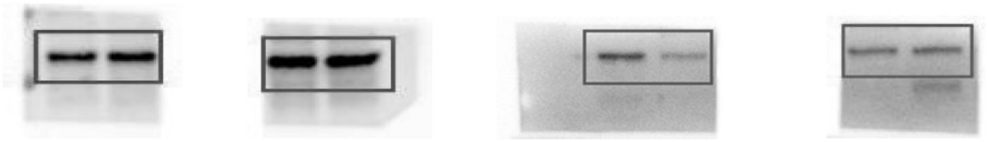

Fig. 3B PGK1

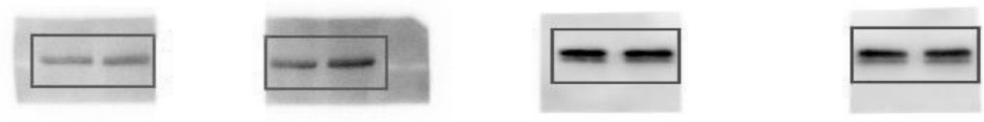

Fig. 3B PKM2

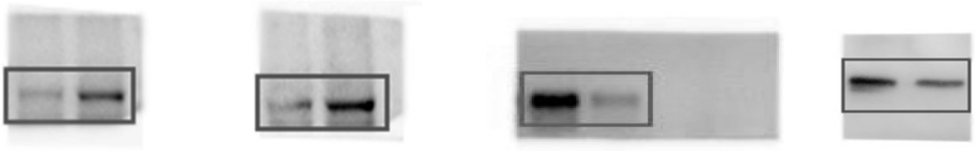

Fig. 3B LDHA

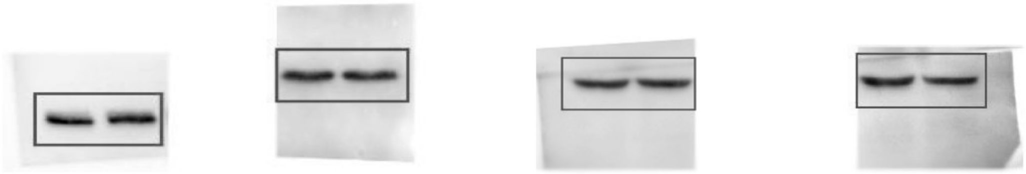

Fig. 3B β-actin

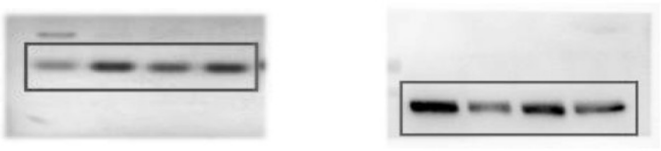

Fig. 4H LDHA

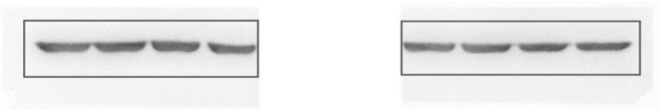

Fig. 4H β-actin

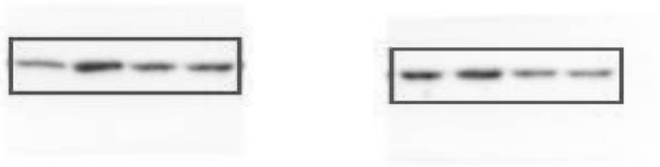

Fig. 8G SHH

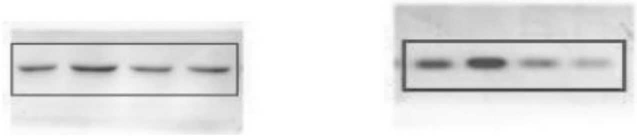

Fig. 8G SMO

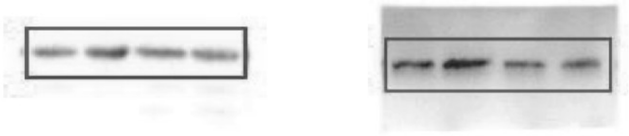

Fig. 8G GLI1

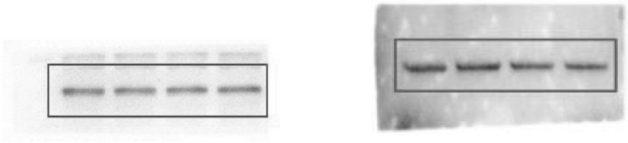

Fig. 8G β-actin
